# Establishment and Characterization of Feline Mammary Tumor Patient-Derived Xenograft Model

**DOI:** 10.3390/ani11082380

**Published:** 2021-08-12

**Authors:** Hsiao-Li Chuang, Yi-Chih Chang, Yi-Ting Huang, Jiunn-Wang Liao, Pei-Ling Kao, Yi-Fei Chen, Bin-Yin Lin, Yi-Lo Lin, Ter-Hsin Chen, Yu-Chih Wang

**Affiliations:** 1 National Laboratory Animal Center, National Applied Research Laboratories, Taipei 106214, Taiwan; p650214@nlac.narl.org.tw; 2 Department of Biotechnology, College of Medical and Health Science, Asia University, Taichung 41354, Taiwan; yichih@asia.edu.tw; 3 Graduate Institute of Veterinary Pathobiology, National Chung Hsing University, Taichung 402, Taiwan; ti123103033@gmail.com (Y.-T.H.); jwliao@dragon.nchu.edu.tw (J.-W.L.); gilliankao@hotmail.com (P.-L.K.); miffy19980416@gmail.com (Y.-F.C.); a0928514860@gmail.com (B.-Y.L.); yllin@dragon.nchu.edu.tw (Y.-L.L.); thc@dragon.nchu.edu.tw (T.-H.C.)

**Keywords:** feline mammary tumor, patient-derived xenograft, cell line

## Abstract

**Simple Summary:**

Feline mammary tumor (FMT) is a relatively commonly diagnosed neoplasm in the cat. Development of new veterinary cancer therapies is limited by the shortage of in vivo models that reproduce tumor microenvironment and metastatic progression. In this study, FMT patient-derived xenografts (PDX) model were established and primary cell line isolated from orthotopic PDX. The tumor grafts conserve original tumor essential features, including distant metastasis. FMT-PDX represents an available resource for bridging the biology of FMT with preclinical studies of FMT in cats.

**Abstract:**

Feline mammary tumor is a relatively commonly diagnosed neoplasm in the cat. Development of new veterinary cancer therapies is limited by the shortage of in vivo models that reproduce tumor microenvironment and metastatic progression. Four feline mammary tumor orthotopic patient-derived xenograft model (PDX) successfully established in NOD-SCID gamma (NSG) mice. The overall success rate of PDX establishment was 36% (4/11). Histological, immunohistochemical, and short tandem repeat analysis showed a remarkable similarity between patient’s tumor and xenograft. The tumor grafts conserve original tumor essential features, including distant metastasis. Primary FMT-1807 cell line isolated from FMT-1807PDX tumor tissue. Tumorigenicity of FMT-1807 cells expanded from PDX was assessed by orthotopic injection into NSG mice. Mice yielded tumors which preserve the lung and liver metastasis ability. This work provides a platform for FMT translational investigation.

## 1. Introduction

Xenograft models using cancer cell lines has been reported to have several limits, such as not reflecting the patient’s drug response sufficiently, limited heterogeneity of the xenograft tumor, and not recapitulating the original tumor microenvironment and tumor–stroma interactions, thus raising concerns that these cell lines may not be fully representative of tumors [1,2,3,4]. Patient-derived xenografts (PDX) have been reported to keep histologic appearance, tumor microenvironments, and molecular fidelity to the original tumor. Therefore, the PDX model is becoming a preferred preclinical tool in the development of effective therapeutics [3,4]. Feline mammary tumors (FMTs) are the frequently diagnosed neoplasm primarily between the ages of 10–12 years old female cats, have high rate of malignancy. The majority of FMTs are highly aggressive, hormone receptor negative cancer, with poor prognosis characterized by early metastasis in female cats [5,6,7]. The standard first-line treatment for FMT is aggressive unilateral or bilateral mastectomy. Based on the aggressive behavior and metastatic rate, adjuvant chemotherapy is often recommended. Chemotherapy is restricted by the severe side effects, repeated requirement for anesthesia and high cost. Although good results can be achieved for some cases, it may not necessarily have an appropriate treatment. The development of more effective and least expensive treatments would be beneficial in clinical settings. The establishment of several FMT cell line has been reported [8,9]. But, established FMT cell lines do not preserve the tumor heterogeneity of the original tumor, likely because of the expansion of a homogenous population over period of time in culture. Despite the potential importance of the PDX model for cancer research and clinical translation, limited studies have reported the engraftment of veterinary PDXs [10,11]. It was reported that FMT-PDX implanted subcutaneously in C.B-17 SCID mice with high engraftment success rate but low metastatic incidence [12]. Different strain of mice, orthotopic and ectopic implantation may influence xenograft success rate and metastatic behavior. The engraftment rate of PDX models will affect the cost of the experiment, the length of the experimental period, and the clinical value of pharmacodynamic tests. The present study aims to establish a bank of serially transplantable, orthotopic, subject-derived FMT grafts in NOD.Cg-*Prkdc^scid^ IL2rg^tm1Wjl^*/SzJ (NSG) mice that retain crucial characteristics of the original tumor specimens.

## 2. Materials and Methods

### 2.1. Primary Tumor Samples

All surgically resected tumor tissue samples were collected after verbal informed consent was received from cat owners at the Nyan cat clinic (*n* = 6), An-Sing animal hospital (*n* = 3) and the veterinary medical teaching hospital of National Chung Hsing University (*n* = 2) under a protocol approved by the Institutional Animal Care and Use Committee (IACUC) of National Chung Hsing University [IACUC Number: 106–73^R3^ and 108–113]. All tumor tissue samples were collected from cat patients without neoadjuvant chemotherapy.

### 2.2. Tissue Implantation of PDXs

NSG mice were purchased from the Jackson Laboratory and bred in the Research Center for Animal Medicine of the National Chung Hsing University. Mice were administered autoclaved food and water ad libitum. There was always a minimum of three mice per passage of tumor engraftment. Anesthetized the mice using isoflurane (AbbVie Global, Taipei, Taiwan) at 4% and an oxygen flow of 1 L/minute (using 4% isoflurane to induce anesthesia in induction box and then 1.5–2% isoflurane for the maintenance with the nose-cone). We implanted a single fragment of fresh tumor (1–8 mm^3^), or injected 1 × 10^6^ primary FMT-1807 cells (isolated from FMT-1807-PDX 3rd passage tumor tissue) in 50 μL Dulbecco’s modified eagle medium (Thermo Fisher Scientific Hyclone, Waltham, MA, USA) mixed with 50 μL Matrixgel Basement Membrane Matrix (BD Biosciences, Bedford, MA, USA) into cleared inguinal mammary fat pads of 6–8-week-old NSG mice. Tumor volumes were calculated weekly using the formula ½ × length × (width)^2^. When tumors reached 500–800 mm^3^, the tumor-bearing mice were isoflurane anaesthetized and PDX tumors were harvested. Donor mice were sacrificed by carbon dioxide asphyxiation. Tumor tissue fragments were transplanted into another three NSG mice, and frozen storage (95% fetal bovine serum + 5% dimethyl sulfoxide) for later use.

### 2.3. Isolation of Primary FMT Cells from PDX Tumor

Primary tumor cell was collected from the surgical specimen during passage (third passage FMT-1807-PDX). Tissue collected from xenograft tumor (FMT-1807-PDX) was minced into <5 mm^3^ pieces and then incubated in PBS with type 3 collagenase (2 mg/mL, Worthington Biochemical Corporation, Lakewood, NJ, USA) and hyaluronidase (10.00 U/mL, Sigma Aldrich, St. Louis, MO, USA) for half hour at 37 °C. After washing with phosphate buffer saline contained 2% of fetal bovine serum, the digested organoids and single cells were then cultured in Dulbecco’s modified eagle medium with 10% fetal bovine serum (FBS; HyClone Laboratories, Logan, UT, USA) at 37 °C with 5% CO_2_. The solid clusters of tumor cells were formed at first passage. After two days, the culture medium was removed and washed with PBS twice. The organoid clusters of tumor cells treated trypsin-EDTA solution 0.25% (Gibco, Grand Island, NY, USA) for 15 min, removed the supernatant and grew the remaining cells with FBS-containing DMEM medium.

Flow cytometric analysis was used to examine the expression of the myofibroblast marker α-smooth muscle actin (α-SMA). Cells (1 × 10^6^) were collected in eppendorf and incubated with the α-SMA (clone 1A4) antibody (Alexa Fluor^®^ 488 Conjugate, eBioscience, San Diego, USA) or Mouse IgG2a Isotype Control (Cell signaling, Danvers, MA, USA) at 37 °C for 30 min in dark, followed by immediate analysis on a BD Accuri™ C6 Plus Flow Cytometer.

Cell proliferation assays were performed to determine growth rates in culture. Cells were seeded in 24-well plate at 1 × 10^5^ cells/well. Cell number was determined with the Luna Automated Cell Counter (Logos Biosystems, Anyang, Korea) daily for a week. Assays were performed in triplicate.

Lentivirus carried the reporter gene encoding red fluorescence protein (RFP; pLAS2w.RFP-C.Ppuro) was purchase from National RNAi Core Facility (Taiwan Academia Sinica). FMT-1807 cells were plated in 6-well dishes at 1 × 10^5^ cells/well and incubated with lentivirus in the presence of polybrene (8 mg/mL) overnight. After 2 days, infected cells (FMT-1807-RFP) were positively selected with puromycin (2 mg/mL) for a week.

To establish tumorigenicity of FMT-1807-RFP cells, 1 × 10^6^ cells from each patient’s cell population was suspended in 100 μL of a 1:1 mixture of DMEM medium and Matrigel Matrix and inoculated into the inguinal mammary fat pads of NSG mice. Imaging was performed using the IVIS Imaging System 200 Series (Xenogen Corporation, Alameda, CA, USA). Photon signal intensity was quantified using Living Image^®^ software (Xenogen Corporation).

### 2.4. Histopathological and Immunohistochemistry Examination

Necropsy was performed after euthanasia by carbon dioxide asphyxiation, and tissues (brain, lung, liver, spleen, kidney, femur) were collected and sectioned to confirm metastasis. Samples were fixed in 4% neutral buffered formalin (femurs decalcified in 10% EDTA solution for 4 weeks), paraffin embedded, sectioned at 4 μm and stained with haematoxylin and eosin. Molecular markers were evaluated immunohistochemically using the avidin-biotin immunoperoxidase method. Sections were deparaffinized by xylene and rehydrated with graded ethanol. Following blocking with 3% hydrogen peroxide and 1% BSA, antigens were retrieved with sodium citrate buffer (10 mM Sodium citrate, 0.05% Tween 20). The slices were incubated with primary antibodies (Table 1) for 1 h at room temperature and secondary antibodies at room temperature for 30 min. Staining with DAB (Vector Laboratories) was applied to the sections. Subsequent slides were counterstained with Gill’s haematoxylin. Expression from immunohistochemical assays was scored by two veterinary pathologists blinded to the origination of the samples. Immunohistochemical semi-quantitative assessment for ER, PR, HER2, CK5/6 or Ki67 were performed following the Allred scoring system guidelines and Soares scoring criteria [12,13].

### 2.5. Short Tandem Repeat (STR) Analysis

To confirm lineage identity of every PDX, STR analysis were performed on different chromosomes at 12 loci [12]. Target DNA (10 ng) was amplified by multiplex polymerase chain reaction using fluorescent dye-linked primers for the 12 loci (F53, C08, B04, G11, SRY, FCA441, D09, F124, C12, C09, F85 and D06) listed in Table 2. Amplification was performed using an TopSTR Amplify PCR Kit (TOPGEN, Taiwan) according to the manufacturer’s instructions. PCR products generated were mixed with Orange 600 DNA size standard (NimaGen, The Netherlands), electrophoresed on an ABI 3730 genetic analyzer (Applied Biosystems, FosterCity, CA, USA), and analyzed with the GeneMapper ID v3.1 software (Applied Biosystems).

### 2.6. Statistical Analysis

All data were expressed as mean ± SD and performed student *t*-test analysis for the independent pairwise samples. All statistical comparisons were carried out using SPSS v.13 software. A two-tailed *p* < 0.05 was considered to represent a statistically significant difference.

## 3. Results

### 3.1. Generation of Tumor Grafts for Feline Mammary Carcinoma

**Figure 1:**
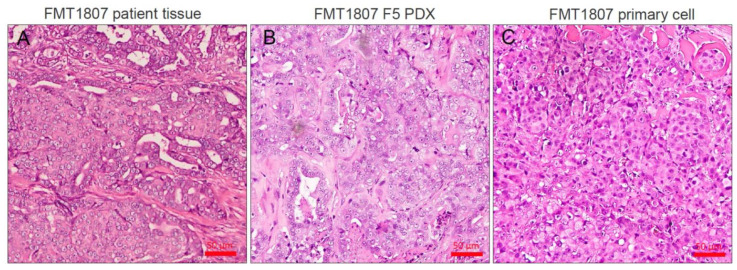
Histological examples of FMT PDXs. H&E staining was used to assess tumor structure of original patient samples (**A**), FMT1807-PDX (**B**) and primary FMT1807 cells implantation (**C**) samples. Bar = 50 μm.

**Figure 2:**
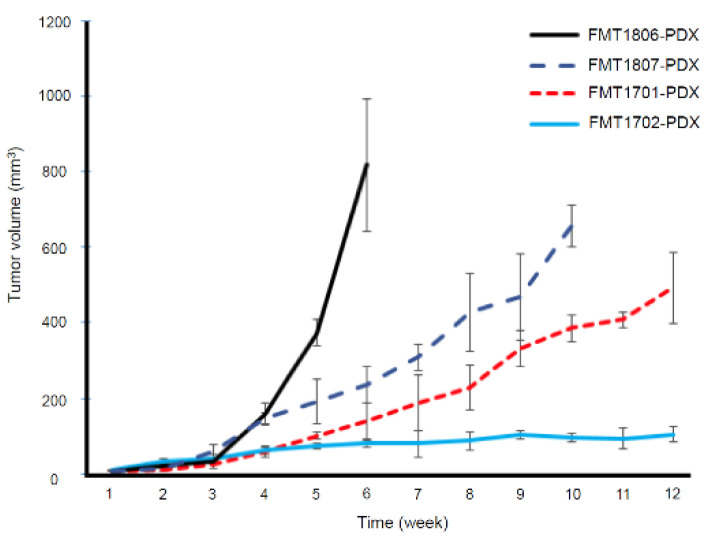
Tumor growth is represented as tumor volume versus time. Six mice were included per group.

We orthotopically transplanted 11 fresh primary FMT samples into mammary fat pads of NSG mice. Tumors grew from 4 out of 11 samples, and we successfully maintained these tumor lines through multiple rounds (at least five passages) of serial transplantation (Table 3). Tumors reached approximately 500–800 mm^3^ volumes prior to collection. Second passage tumors showed similar overall growth rate to the initial founders with a >90% successful engraftment and tumor growth rate. After histological review of all successful cases, we found that the resultant tumor grew from PDX models displayed histological preservation to the original patient, characterized by cuboidal to columnar epithelial cells that formed irregular tubular structures (Figure 1A,B), more gland-like characteristics than the tumor growth from primary FMT-1807 cells transplantation (Figure 1C). Nucleolar anisokaryosis was repressed in primary tumor cells. Two tumor grafts (FMT-1701 and FMT-1807) were triple negative basal-like FMT, one tumor graft was luminal A (FMT-1702), another one was HER2-positive (FMT-1806), according to immunohistochemical analysis (Table 1). Tumor growth parameters of each FMT-PDX are shown in Figure 2.

**Figure 3:**
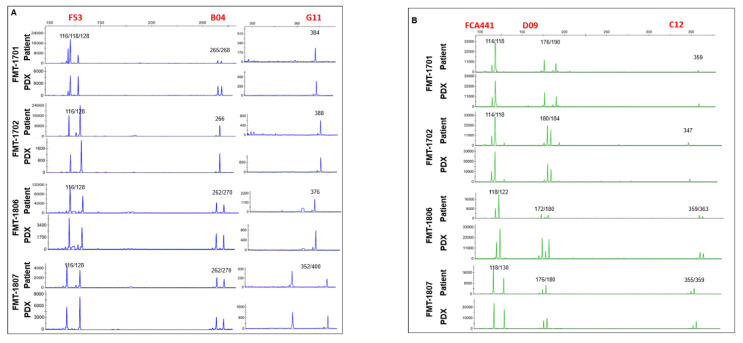

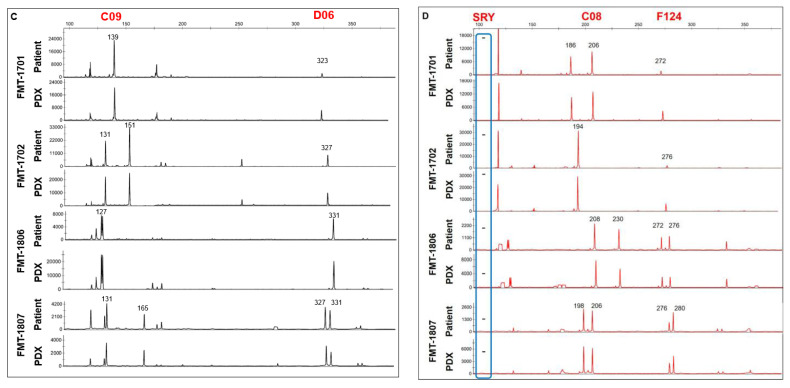
STR amplification of FMTs. Four nanograms of extracted DNA from patient cat tissues or PDX graft tissues were performed cat STR typing. The current dye labels used for the assay include 6-FAM (blue; **A**), VIC (green; **B**), NED (black; **C**) and ROX (red; **D**). X axis show the allele size, and the Y axis show the intensity.

We performed microsatellite STR analyses to determine whether the established PDX model retained the identical pattern of the original tumors. We performed PCR using STR markers between tumor tissues from patients and from mice that occurred in established PDX models. As a result, the expression levels of STR markers were observed at the same chromosome loci in parental tumor tissues from patients and fifth passage PDX model (Figure 3). These results showed that the patient and tumor xenografted mouse models preserved the same STR profiles until at least fifth-generation.

**Figure 4:**
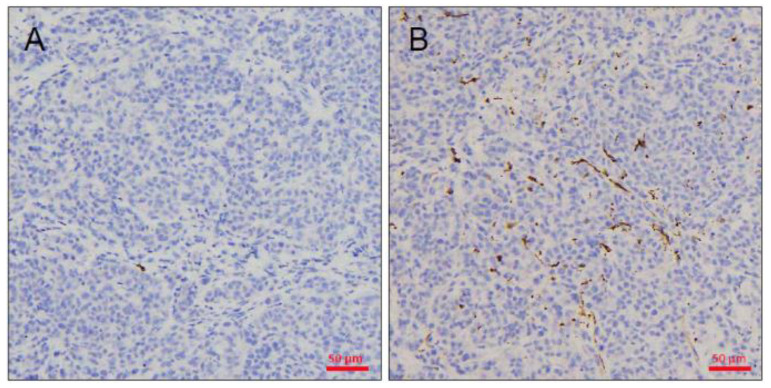
To confirm lymphomatous transformation in PDX tumors, immunohistochemical staining for analysis of CD3 (**A**), and CD20 (**B**) expression were performed. The tumor cells were CD3-negative and CD20-negative, indicating there were no lymphomatous transformation in PDX tumors. Bar = 50 μm.

In human studies, lymphoma transformation was reported in some PDX cases. After histological review of all successful cases, we found that no lymphomatous transformation or lymphocytic dominant pattern in tumor tissues obtained from mice in tumors stained for anti-CD20 (Figure 4A) or anti-CD3 (Figure 4B) antibodies.

### 3.2. Tumor Grafts Emulate Metastasis Seen in PDX Mice

**Figure 5:**
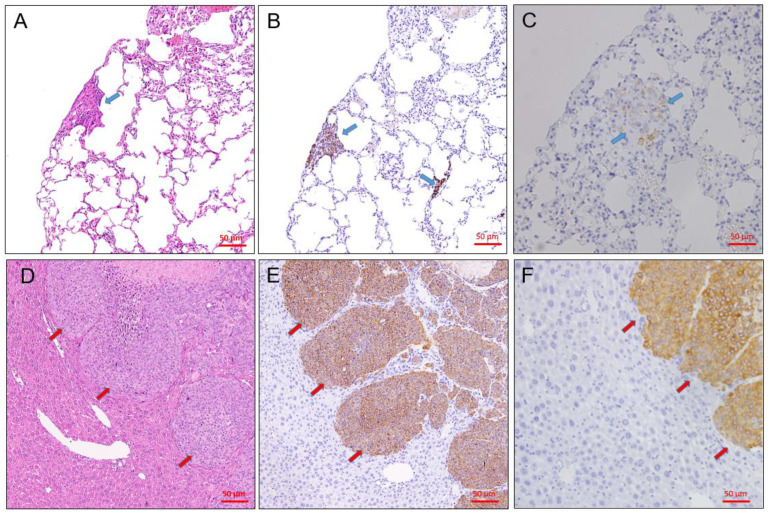
Representative examples of spontaneous metastases from FMT1807-PDX as detected in sections of lung (**A**–**C**) and liver (**D**–**F**) of mice at necropsy. We identified metastases by H&E staining (**A**,**D**) or by staining with antibodies specific to vimentin (**B**,**E**) and pan-cytokeratin (pan-CK; **C**,**F**). The arrows indicate the metastatic lesion. Bar = 50 μm.

Tumor metastases in these PDX lines were identified in the lung, liver, spleen, kidneys, and lymph nodes and were examined at the time of necropsy. Tumor metastases were detected grossly, by H&E staining and immunohistochemical staining. We detected spontaneous metastasis in the axillary node, lung, and liver of mice with tumor grafts (Table 1). All of the metastatic tumors were confirmed as carcinomas by vimentin and pan-cytokeratin (pan-CK) IHC staining (Figure 5). Metastasis frequencies of FMT-1806 PDX and FMT-1807 PDX were 83.3% (5/6) and 100% (6/6), respectively (Table 4).

### 3.3. Immunohistochemical Characteristics of PDX Tumors

**Figure 6:**
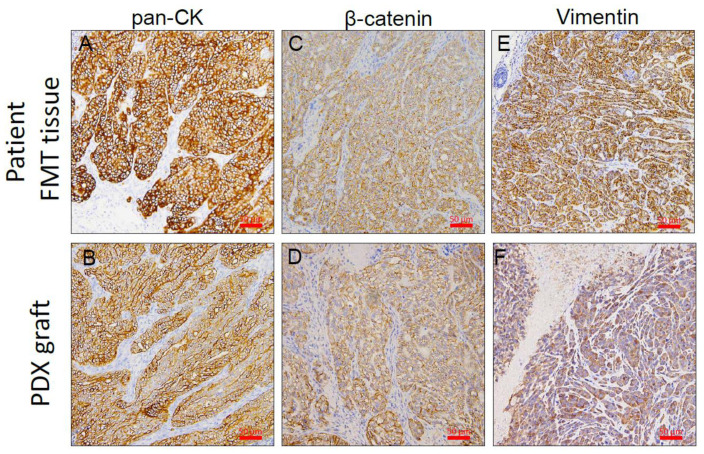
A representative PDX graft (FMT1807) is shown in comparison to the original sample. Sections from the primary FMT of patient cat and from representative PDX grafts from the same individual are shown. Shown are antibody stains for pan-cytokeratin (pan-CK; **A**,**B**), β-catenin (**C**,**D**) and vimentin (**E**,**F**). Positive antibody signals are shown in brown, and the hematoxylin counterstain is shown in blue. Bar = 50 μm.

We analyzed the major molecular characteristics (pan cytokeratin, β-catenin and vimentin) of original tumors and PDX grafts following five passages in mice (Figure 6). The expressions of these molecular markers revealed similarities between the xenograft tumors and the patients’ tumor samples showed that the PDX model retained not only the histology but also the expression of cytological characteristics was preserved.

### 3.4. Establishment of Primary FMT Cells from PDX

**Figure 7:**
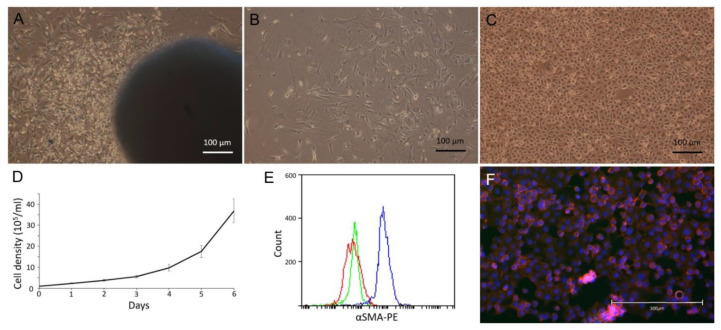
Isolation of primary FMT cells from PDX tumor. (**A**) Mixed primary culture (organoid) derived from FMT-1807PDX xenograft tumor. (**B**) Mesenchymal cells derived by differential trypsinization from FMT-1807PDX xenograft tumor, cultured in DMEM supplemented with 10% FBS. (**C**) Morphology of primary FMT-1807 cells after differential trypsinization. (**D**) In vitro growth kinetics of FMT-1807 cells. (**E**) Flow cytometric analysis of α-SMA expression of primary FMT-1807 cells (red) and mesenchymal cell derived by differential trypsinization (blue). Isotype control (green) indicates staining of cells with the isotype control antibody. (**F**) Confocal image of purified RFP-tagged FMT-1807 cells.

**Figure 8:**
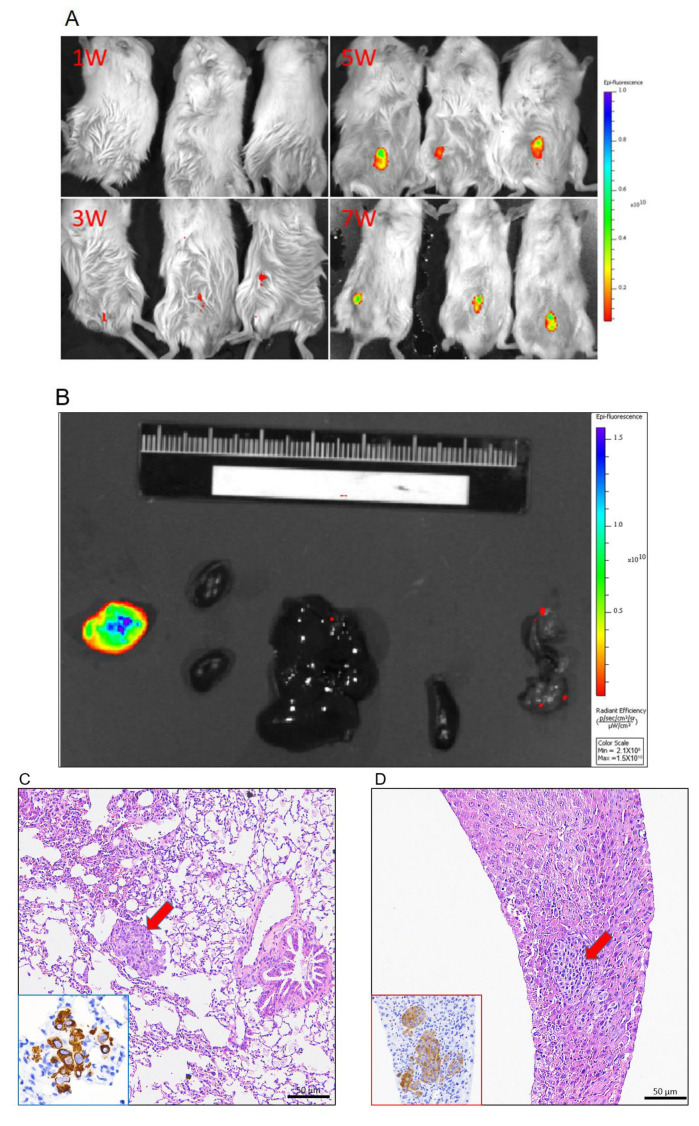
Analysis of NSG mice engrafted withFMT-1807-RFP cells. (**A**) Optical imaging of mice bearing tumors induced by FMT-1807-RFP cells into the inguinal mammary fat pads monitored via ex vivo IVIS imaging. The time course is indicated. (**B**) Tumor spontaneous metastasis to organs monitored via IVIS imaging (from left to right: kidney, liver, spleen and lung). Histopathology of metastatic tumors in mouse models with FMT-1807-RFP cells. H&E staining and IHC staining for vimentin of paraffin sections of lung (**C**) and liver metastases (**D**). The arrows indicate the metastatic lesion.

Primary FMT-1807 cell line was isolated from FMT-1807PDX tumor formed adherent monolayers in culture (Figure 7A–C) with fast growth kinetics (Figure 7D). Flow cytometric analysis was used to examine the depletion of fibroblasts through differential trypsinization. Cultured FMT-1807 cells were negative for α-SMA, indicating the absence of fibroblastic cells (Figure 7E). To confirm tumorigenicity of FMT-1807 cells, the ability of FMT-1807-RFP cells (Figure 7F) to form tumors in NSG mice was determined. FMT-1807-RFP cells were able to grow in vivo (Figure 8A). In addition, metastasis to lung and liver were detected in tumor-bearing animals as shown by IVIS (Figure 8B) and vimentin staining (Figure 8C,D).

## 4. Discussion

In the present study, we established a bank of serially transplantable, orthotopic mammary tumor grafts that retained critical features of the original tumor specimens from cats with FMC. The result shows that the FMT-PDX grafts maintain key features of the original tumors, including histopathology and immunohistochemical markers. The establishment of several FMT cell lines has been reported [6,11], but only few reports about feline PDX [11]. The take rate of FMT-PDX is higher than other type cat cancer [11,21], canine tumor [22,23] or human tumor [24], it might be suggested that high malignancy of FMT. Lower metastasis incidence found in ectopic FMT-PDX [11] suggested that the preservation of the tumor microenvironment in subcutaneous xenograft models may differ from orthotopic FMT implantation.

Animal models are useful in vivo tools for the molecular studies of tumor progression and metastasis. One of the important features between the FMTs in patient cats and their corresponding PDX grafts is their ability to spontaneously metastasize. To create an effective translational model, we isolated purified populations of FMT cells in culture derived from our FMT-1807PDX model murine xenograft. FMT cell line is important resource for studying cancer cell biology, as well as for developing new strategies against cancer cell validation in vitro and in vivo. Numerous FMT cell lines have been reported and characterized [6,11]. Most of the previously established FMT cell lines used in research were derived from primary or metastasis FMTs, whereas our new cell lines were derived from murine patient tumor xenografts. We have found this approach to be beneficial due to the relatively larger amounts of tumor tissue from which to culture cells, as primary patient tumor specimens are usually more limited in volume. Also, murine xenografts are potentially endlessly expandable, so it is possible to isolate new cell lines repeatedly after the original tumor resection has taken place. The primary FMT-1807 cell has demonstrated efficient tumorigenicity when orthotopic injected into NSG mice. Therefore, this approach can be used to enables concurrent study of patient tumor xenografts and their derived cell line to be experimented with both in vitro and in vivo.

## 5. Conclusions

In summary, a PDX model of FMT was developed in NSG mice. The FMT-PDX model is relatively easy to carry out and may serve as a platform for further cancer research and translational work.

## Figures and Tables

**Table 1:** Antibodies applied for the immunohistochemical examination.

Target	Clone	Dilution	Manufacturer	Reference
ER	6 F11	1:100	Thermo Scientific	Burrai et al., 2010 [14]
PR	10A9	1:50	Meridian Life Science	Burrai et al., 2010 [14]
HER2	CB11	1:200	Invitrogen	Soares et al., 2016 [13,15]
Pan-cytokertin	AE1/AE3	1:100	Dakocytomation	Scott et al., 2011 [16]
β-catenin	14	1:100	BD Biosciences	Zappulli et al., 2012 [17]
Vimentin	V9	1:100	Dakocytomation	Peñafiel-Verdu et al., 2012 [18]
CD3	SP7	1:50	Abcam	Furukawa et al., 2017 [19]
CD20	B-Ly1	1:100	Santa Cruz Biotechnology	Darbès et al., 1997 [20]
Cytokeratin 5/6	D5/16B4	1:100	Sigma-Aldrich	Soares et al., 2016 [13,15]
Ki-67	polyclonal	1:500	Thermo Scientific	Soares et al., 2016 [13,15]

**Table 2:** Cat multiplex primer sequences and final concentrations.

STR Marker	Concentration (uM)	Dye	Primer Sequence
F53	0.9	6FAM	Forward: CCTATGTTGGGAGTAGAGATCACCT
	0.9		Reverse: GTGTCTTGAGTGGCTGTGGCATTTCC
C08	0.9		Forward: GATCCATCAATAGGTAAATGGATAAAGAAGATG
	0.9	ROX	Reverse: TGGCTGAGTAATATTCCACTGTCTCTC
B04	0.9	6FAM	Forward: TGAAGGCTAAGGCACGATAGATAGTC
	0.9		Reverse: GTGTCTTCCACCCAGGTGTCCTGCTTC
G11	1.44	6FAM	Forward: ATCCATCTGTCCATCCATCTATT
	1.44		Reverse: GGTCAGCATCTCCACTTGAGG
SRY	0.04	ROX	Forward: TGCGAACTTTGCACGGAGAG
	0.04		Reverse: GCGTTCATGGGTCGTTTGACG
FCA441	0.6		Forward: GTGTCTTGATCGGTAGGTAGGTAGATATAG
	0.6	VIC	Reverse: ATATGGCATAAGCCTTGAAGCAAA
D09	0.16	VIC	Forward: CCGAGCTCTGTTCTGGGTATGAA
	0.16		Reverse: GTGTCTTTCTAGTTGGTCGGTCTGTCTATCTG
F124	0.6	ROX	Forward: TGTGCTGGGTATGAAGCCTACTG
	0.6		Reverse: GTGTCTTCCATGCCCATAAAGGCTCTGA
C12	0.6	VIC	Forward: GAGGAGCTTACTTAAGAGCATGCGTTC
	0.6		Reverse: GTGTCTTAAACCTATATTCGGATTGTGCCTGCT
C09	1.2	NED	Forward: AAATTTCAATGTCTTGACAACGCATAAG
	1.2		Reverse: GTGTCTTCCAGGAACACCATGTTGGGCTA
F85	1.44	NED	Forward: TAAATCTGGTCCTCACGTTTTC
	1.44		Reverse: GCCTGAAAATGTATCCATCACTTCAGAT
D06	1.2	NED	Forward: CCAAGGAGCTCTGTGATGCAAA
	1.2		Reverse: GTTCCCACAGGTAAACATCAACCAA

**Table 3:** Data for each tumor case and corresponding FMT-PDX line.

				Subject Information						Xenograft Information				
Case	Breed	Tumor Grade	Histological Type	Histological Grade ^a^	ER	PR	HER2	Molecular Subgroup	Prognosis	ER	PR	HER2	Duration in First Passage (weeks) ^b^	Metastasis
FMT-1701	Mixed	T1N0M0	Tubulopapillary	II	-	-	-	Triple negative basal-like	Not available	-	-	-	12–14	Not detected
FMT-1702	Mixed	T1N0M0	Tubulopapillary	I	+	-	-	Luminal A	Not available	+	-	-	>6 month	LN
FMT-1806	American shorthair	T3N0M0	Solid	III	-	-	2+	HER2-positive	Rrcurrence, lung metastasis	-	-	2+	6–7	Lung
FMT-1807	Mixed	T2N0M0	Tubulopapillary	III	-	-	-	Triple negative basal-like	Rrcurrence, lung metastasis	-	-	-	10–11	Lung, liver

^a^ According to the Elston and Ellis histologic grading system. ^b^ Interval between transplantation and subpassage to passage 2 (>500 mm^3^). LN, lymph node.

**Table 4:** Tumor growth parameters of female mice xenografted with FMT-1806PDX or FMT-1807PDX.

PDX	% of Tumor Growth	Time of 500 mm^3^ Volume (days)	% of Animals with Metastasis
FMT-1806PDX P3 (*n* = 6)	100	34.2 ± 2.4	83.3% Lung (5/6)
FMT-1807PDX P2 (*n* = 6)	100	56.5 ± 3.2	100% Lung (6/6), liver (2/6)

## Data Availability

The datasets used and/or analyzed during the current study are available from the corresponding author on reasonable request.
